# Elucidation of the CCR1- and CCR5-binding modes of MIP-1α by application of an NMR spectra reconstruction method to the transferred cross-saturation experiments

**DOI:** 10.1007/s10858-015-9992-x

**Published:** 2015-10-15

**Authors:** Chie Yoshiura, Takumi Ueda, Yutaka Kofuku, Masahiko Matsumoto, Junya Okude, Keita Kondo, Yutaro Shiraishi, Ichio Shimada

**Affiliations:** Graduate School of Pharmaceutical Sciences, The University of Tokyo, Hongo, Bunkyo-ku, Tokyo 113-0033 Japan; Precursory Research for Embryonic Science and Technology, Japan Science and Technology Agency, Chiyoda-ku, Tokyo 102-0075 Japan; Japan Biological Informatics Consortium, Aomi, Koto-ku, Tokyo 135-8073 Japan

**Keywords:** Membrane proteins, G-protein coupled receptors, Transferred cross-saturation, Nanodiscs, Sparse sampling, Surface plasmon resonance

## Abstract

C–C chemokine receptor 1 (CCR1) and CCR5 are involved in various inflammation and immune responses, and regulate the progression of the autoimmune diseases differently. However, the number of residues identified at the binding interface was not sufficient to clarify the differences in the CCR1- and CCR5-binding modes to MIP-1α, because the NMR measurement time for CCR1 and CCR5 samples was limited to 24 h, due to their low stability. Here we applied a recently developed NMR spectra reconstruction method, Conservation of experimental data in ANAlysis of FOuRier, to the amide-directed transferred cross-saturation experiments of chemokine receptors, CCR1 and CCR5, embedded in lipid bilayers of the reconstituted high density lipoprotein, and MIP-1α. Our experiments revealed that the residues on the N-loop and β-sheets of MIP-1α are close to both CCR1 and CCR5, and those in the C-terminal helix region are close to CCR5. These results suggest that the genetic influence of the single nucleotide polymorphisms of MIP-1α that accompany substitution of residues in the C-terminal helix region, E57 and V63, would provide clues toward elucidating how the CCR5–MIP-1α interaction affects the progress of autoimmune diseases.

**Electronic supplementary material:**

The online version of this article (doi:10.1007/s10858-015-9992-x) contains supplementary material, which is available to authorized users.

## Introduction

Chemokine receptors are members of G protein-coupled receptor (GPCR) superfamily and regulate various inflammation and immune responses. C–C chemokine receptor 1 (CCR1) and CCR5 are chemokine receptors expressed in monocytes, macrophages, and Th1 cells and recognize C–C type chemokines, such as MIP-1α, MIP-1β, and RANTES (Devalaraja and Richmond 1999). CCR1 and CCR5 are therapeutic targets for autoimmune diseases (O’Hayre et al. 2010; Proudfoot et al. 2010), multiple sclerosis (MS) and rheumatoid arthritis. CCR1 and CCR5 regulate the progression of these diseases differently (Mahad et al. 2004), and thus the elucidation of the roles of CCR1 and CCR5 would provide clues for understanding the mechanisms underlying the progression of these autoimmune diseases.

We embedded CCR5 into reconstituted high density lipoprotein (rHDL), which enabled CCR5 to maintain its functions for ~24 h and thus to be suitable for structural analyses (Yoshiura et al. 2010). The methyl-directed transferred cross-saturation (TCS) experiments of the complex revealed that valine 59 and valine 63 of MIP-1α are in close proximity to CCR5 in the complex (Yoshiura et al. 2010; Ueda et al. 2014). However, the number of residues identified at the binding interface was not sufficient to clarify the differences in the CCR1- and CCR5-binding modes to MIP-1α, although a crystal structure of CCR5 that was engineered to stabilize the inactive state and bound to an inverse agonist and two crystal structures of other chemokine–chemokine receptor complexes, CXCR4–vMIPII and US28–CX3CL1, are available (Tan et al. 2013; Burg et al. 2015; Qin et al. 2015).

We recently developed an NMR spectra reconstruction method, “Conservation of experimental data in ANAlysis of FOuRier” (Co-ANAFOR), to reconstruct the crowded spectra from the sparsely sampled time-domain data (Ueda et al. 2015). The number of sampling points required for the TCS experiments of membrane proteins, photosystem I and cytochrome *b*_*6*_*f*, and their ligand, plastocyanin, with Co-ANAFOR was half of that needed for linear prediction, and the peak height reduction ratios of the spectra reconstructed from truncated time-domain data by Co-ANAFOR were more accurate in our hands than those reconstructed from non-uniformly sampled data by compressed sensing (Ueda et al. 2015).

Here, the reconstruction of the truncated time-domain data by Co-ANAFOR was applied to amide-directed TCS experiments, which enabled the identification of the total binding interface for analyses of the interactions between chemokine receptors, CCR1 and CCR5, and their ligand, MIP-1α.

## Materials and methods

### Preparation of the [ul-^2^H,^15^N] MIP-1α variant

The previously prepared MIP-1α variant (P8A/F13Y/E67Q) expression plasmid (Yoshiura et al. 2010) was transformed into *Escherichia coli* strain BL21 Origami B (DE3) (Novagen). Cells were grown at 37 °C in 10 mL of Luria–Bertani medium for 12 h, and were then harvested and resuspended in fresh M9 minimal medium. Uniformly ^2^H- and ^15^N-labeled proteins for TCS experiments were produced in 99.8 % ^2^H_2_O M9 minimal medium with 1 g/L ^15^NH_4_Cl and 2 g/L [^2^H_7_]-glucose. Protein expression was induced by the addition of isopropyl-β-d-thiogalactopyranoside to a final concentration of 0.1 mM when the culture reached an OD_600_ of 0.7–0.9. After shaking incubation at 25 °C for 20 h, the cells were pelleted by centrifugation at 7000×*g* for 10 min. The MIP-1α variant was prepared as described previously (Yoshiura et al. 2010).

### Preparation of CCR1 and CCR5 with the insect expression system

The cDNA fragment encoding human CCR1 was amplified by PCR, and was cloned into the pFastBac1 vector (Invitrogen). A DNA fragment, encoding with the N-terminal gp64 promoter and gp64 signal sequence and the C-terminal eight residues of a linker and nine residues (TETSQVAPA) of bovine rhodopsin was introduced just before the stop codon of the CCR1 gene, as the 1D4 antibody epitope tag (1D4-tag). This plasmid is referred to as pFastBac1-CCR1-1D4. Recombinant baculoviruses were generated using the pFastBac1-CCR1-1D4 plasmid and the Bac-to-Bac Baculovirus Expression System (Invitrogen), according to the manufacturer’s instructions. Recombinant baculoviruses for CCR5 expression were prepared as described previously (Yoshiura et al. 2010).

For the large scale expression of CCR1 and CCR5, express SF+ cells (Protein Sciences Corp.) were grown in 1–3 L of Sf900-II serumfree media (Invitrogen) at 27 °C, using 250 mL Erlenmeyer flasks (Corning). When the cells reached a density of 2.0 × 10^6^ cells/mL, the high titer virus stock (10 per 100 mL of cells) and 1.25 μg/mL of E64 (Peptide Institute) were added, and the cells were harvested 48 h post infection. The cells were lysed by sonication and subjected to discontinuous sucrose density gradient ultracentrifugation to purify the membrane fraction, as described previously (Yoshiura et al. 2010).

### Preparation of CCR1-rHDL and CCR5-rHDL

The CCR1 or CCR5-containing membrane fraction, prepared from 0.8 L culture of SF+ cells, was solubilized with ~10 mL of HBSG buffer (20 mM HEPES, 150 mM NaCl, 20 % glycerol, pH 7.8) containing 1 % DDM and a protease inhibitor cocktail (Nacalai Tesque). After a 20 min incubation at 4 °C, ~8 mg of MSPE3 was added to achieve a final concentration of ~40 μM. The reconstitution mixture was incubated for 10 min at 4 °C, and the self-assembly process was initiated by adding ~1 g of Bio-Beads SM-2 (Bio-Rad). The mixture was incubated with the beads for 1 h at 4 °C with gentle agitation, and the beads were then removed. The addition and removal of the beads were repeated twice.

The reconstitution mixture was mixed with 0.5 mL 1D4-Sepharose, in which 5 mg/mL of the 1D4 antibody (University of British Columbia) was coupled to CNBr-activated Sepharose 4B (GE Healthcare). The mixture was incubated for 1.5 h at 4 °C, and the supernatant was removed after centrifugation. The beads were washed three times with 10 mL of HBSG. For the elution of CCR1- or CCR5-rHDL, the column was incubated for 30 min at 4 °C in the presence of 0.5 mL of HBSG, containing 0.4 mg/mL of the nonapeptide (TETSQVAPA), and the supernatant was collected by centrifugation. This elution process was repeated four times. The eluates were mixed with 1 mL of Ni-affinity chromatography column resin equilibrated with the HBSG buffer, and incubated for 2 h at 4 °C. The column was then washed with 10 mL of HBSG buffer and 10 mL of TCS buffer (20 mM HEPES, 100 mM NaCl, 5 % glycerol, 75 % D_2_O, pH 6.0). The rHDL was eluted with 3 mL of TCS buffer containing 100 mM imidazole. The elution fractions containing CCR1- or CCR5-rHDL were concentrated to 0.25 mL with an Amicon Ultra 0.5 filter (MWCO 30 K, Millipore), after the removal of the imidazole.

The yield and purity of the prepared CCR1- or CCR5-rHDL were analyzed by 15 % SDS-PAGE. Gels were stained with a Silver Staining Kit (Daiichi Pure Chemicals).

### Guanine nucleotide exchange assay

The heterotrimeric G protein was prepared as described previously (Yoshiura et al. 2010). The purified CCR1-rHDL was incubated with the G proteins and the MIP-1α variant (P8A/F13Y/E67Q), in a final volume of 0.3 mL of buffer (20 mM HEPES, pH 7.8, 100 mM NaCl, 10 mM MgCl_2_, and 10 μM GDP). The reaction mixtures were first incubated for 1 h at 4 °C and subsequently for 30 min at room temperature with gentle agitation, and then a 30 μL aliquot of non-hydrolyzable GTP modified with fluorescent Europium (Eu-GTP, PerkinElmer) was added to a final concentration of 10 nM. The reaction mixtures were further incubated for 30 min at room temperature (~298 K), and the reaction was terminated by the addition of non-labeled GTPγS (PerkinElmer) to a final concentration of 5 μM. To capture the Gα subunit with its N-terminal His-tag, each mixture was combined with 120 μL of the equilibrated Ni affinity beads, and was diluted with buffer to a final volume of 0.75 mL. After a 1.5 h incubation, the beads were applied to three wells in an AcroWell filter plate (Pall Corporation). To remove the unbound Eu-GTP, the beads were washed twice by filtration through the filters with 0.25 mL of the HBSG buffer. The bound Eu-GTP was measured, using an Envision 2103 Multilabel Reader (PerkinElmer), with excitation at 320 nm and emission measured at 620 nm.

### SPR analysis

The MIP-1α variant binding activity of CCR1-rHDL was analyzed using a BIAcore T-100 instrument (GE Healthcare Life Sciences). The 1D4 antibodies were first immobilized on the sensor chip CM5 using standard amine-coupling chemistry, resulting in a signal of ~12,000 resonance units. CCR1-rHDL was captured on the sensor chip via the 1D4 antibodies, at a flow rate of 1 μL/min and at 4 °C, resulting in a signal of ~3000 resonance units. The empty-rHDL, composed of MSPE3-1D4 and the lipids derived from Sf+ cells, was captured on the control flow cell under the same conditions, resulting in a signal of ~3000 resonance units. The flow cells were washed ten times with injections of 10 μL of running buffer (20 mM HEPES, pH 7.2, 100 mM NaCl), at a flow rate of 30 μL/min.

The binding assay was performed in running buffer, at a flow rate of 30 μL/min and at 25 °C, using serial dilutions of the MIP-1α variant in the 0.13–5.0 μM range. The dissociation constant was obtained from the steady-state curve fitting analysis, using the Biacore T100 Evaluation Software (GE Healthcare Life Sciences).

### TCS experiments with MIP-1α, CCR1, and CCR5

The methyl-directed TCS experiments were performed with 10 μM (final concentration) of [u-^2^H, (Ile-, Leu-, Val-^13^C^1^H_3_)] MIP-1α variants and about 1/10 molar equivalent of CCR1-rHDL, as described previously (Yoshiura et al. 2010). In the amide-directed TCS experiments, the lyophilized [u-^2^H, ^15^N]-MIP-1α variants (50 μM final concentration) were combined with about 3/50 molar equivalent of CCR1-rHDL or CCR5-rHDL, in 20 mM HEPES, pH 6.0, containing 100 mM NaCl, 75 % D_2_O, and 5 % (v/v) glycerol. For negative control experiments, the same experiments were performed with the sample including the empty-rHDL, composed of the MSPE3 and lipids derived from Sf+ cells. All of the amide-TCS spectra were recorded at 15 °C with a Bruker Avance 800 spectrometer equipped with a cryogenic probe, using the pulse scheme described previously (Yoshiura et al. 2010). The irradiation frequency was set at 1.9 ppm, and the maximum radio frequency amplitude was 0.21 kHz for WURST (the adiabatic factor Q_0_ = 1). The irradiation time and the additional relaxation time were set to 1 and 4 s, respectively. The maximum evolution times in the ^1^H and ^15^N dimensions before the insertion were 39.9 and 12.3 ms, respectively. The spectra with and without irradiation and two spectra with different irradiation times were recorded for each sample with 512 × 24 complex points, and 64 scans/FID gave rise to an acquisition time of ~24 h.

The reconstruction by Co-ANAFOR were performed by in-house developed programs written in a programming language, Python 2.7, supplemented with extension modules, Numpy 1.5, Scipy 0.9, and l1l2py (http://slipguru.disi.unige.it/Software/L1L2Py/). The Tikhonov regularization factor and the linewidth of the signals in the directly observed dimension, which were utilized in the reconstruction by Co-ANAFOR, were set to 0.01 and ±0.02 ppm, respectively. The relaxation rates of the signals, utilized for the calculation of the inserted data, were uniformly set to the reciprocal of the maximum evolution time after the insertion of the calculated data. The reconstruction by LP was performed by Topspin 3.1 (Bruker Biospin). The number of coefficients in LP was set to 8. The square sine with a 90° phase shift window function was manipulated after the insertion.

## Results

### Application of Co-ANAFOR to the TCS experiments with the MIP-1α variant and CCR5-rHDL

Amide-directed TCS experiments of CCR5-rHDL and MIP-1α were performed with a sample containing ~3 μM non-labeled CCR5 and an excess amount (50 μM) of a [u-^2^H, ^15^N]-labeled MIP-1α variant, which is monomeric and retains the agonist activity for CCR5 (Yoshiura et al. 2010). Due to the low stability of CCR5, even in the lipid bilayer environment of rHDL, the NMR measurement time for this sample was limited to 24 h, and thus over 24 complex data could not be experimentally observed with a sufficient S/N ratio.

**Fig. 1:**
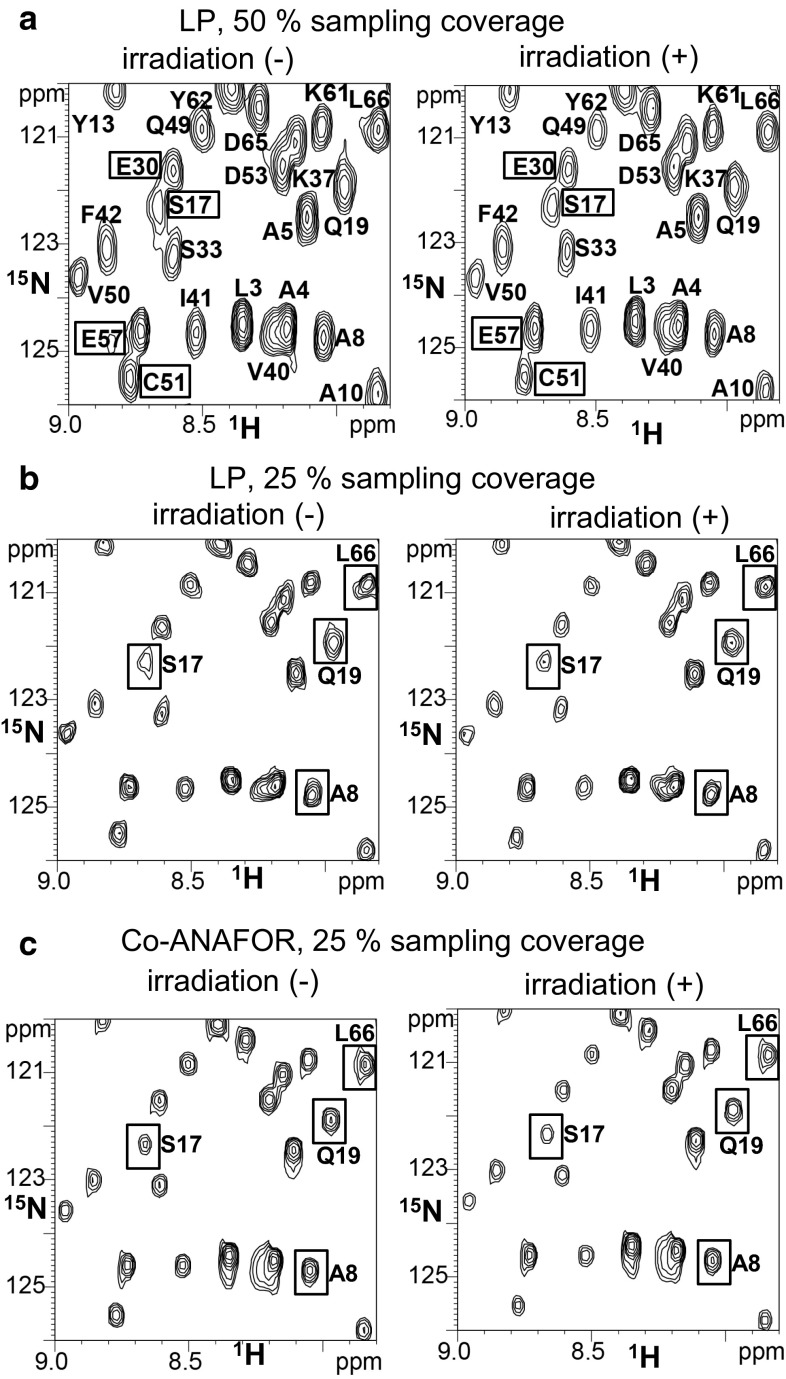
Reconstruction of TCS spectra of MIP-1α and CCR5-rHDL. **a**–**c** [^1^H,^15^N] TROSY spectra observed at 18.8 T for an excess amount of [ul-^2^H, ^15^N] MIP-1α relative to the CCR5-rHDL, with or without irradiation. **a** Spectra from 24-point experimentally observed data and 24-point data inserted for reconstruction by LP. **b**, **c** Spectra from 24-point experimentally observed data and 72-point data inserted for reconstruction by LP (**b**) or Co-ANAFOR (**c**). For lineshape clarity, the signals are not labeled in **b** and **c**. In **a**, S17, E30, C51, and E57, which exhibited signal overlap, have labels enclosed in *boxes*. In **b**, **c**, resonances with lineshapes in the spectra without irradiation that are remarkably different from those with irradiation in (**b**) are labeled and enclosed in *boxes*

In the spectra from the experimentally recorded data and the 24-point data inserted for the reconstruction by LP (Fig. 1a), the resonances from S17 and C51 overlapped with those from E30 and E57, respectively. In the spectra without irradiation from the 24-point experimentally recorded data and the 72-point data inserted for the reconstruction by LP, the lineshapes, as well as the intensities, of the signals were different from those of the spectra with irradiation (Fig. 1b). These lineshape differences would cause errors in the intensity reduction ratios in the spectra reconstructed by LP. In our previously reported spectra of the TCS experiments of membrane proteins, photosystem I and cytochrome *b*_*6*_*f*, and plastocyanin reconstructed by LP with sampling coverages identical to those of Fig. 1b, the lineshapes of the signals with irradiation were also remarkably different from those without irradiation, and the peak height reduction ratios of the signals were significantly different from those of the unreconstructed spectra with 100 % sampling coverage (Ueda et al. 2015). These results suggest that the peak height ratios of the spectra in Fig. 1b would be different from those of the unreconstructed spectra with 100 % sampling coverage, although the latter are not available, due to the low stability of CCR5. However, the signal overlap and distortion were not observed in the spectra from the experimentally recorded data and the 72-point data inserted for the reconstruction by Co-ANAFOR (Fig. 1c).

**Fig. 2:**
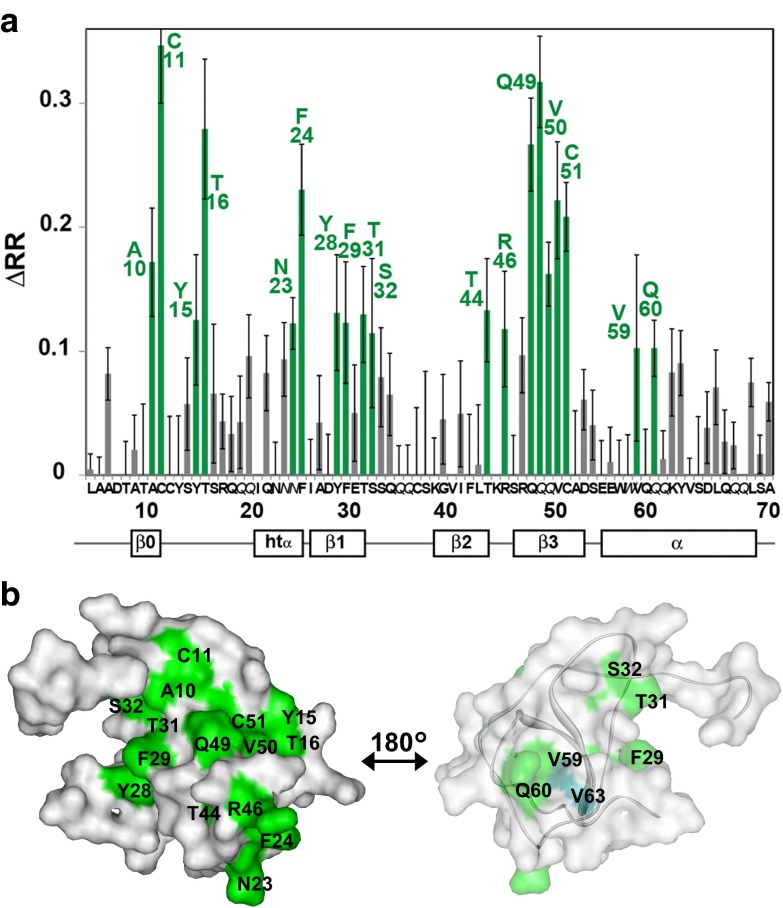
CCR5-binding site on MIP-1α determined with the TCS method along with Co-ANAFOR. **a** Plot of the difference in the reduction ratio (ΔRR) signal intensities originating from the amide groups in the amide-directed TCS experiments with MIP-1α and CCR5-rHDL. Cross-peaks with ΔRR > 0.1 and <0.1 are *colored green* and *gray*, respectively. The *error bars* represent the experimental errors, calculated from the root sum square of (noise level/signal intensity) in the four spectra, with and without irradiation in the main and control experiments. Sidechains are denoted in *italics*. **b** Mapping of the affected residues in the TCS experiments with CCR5-rHDL on the MIP-1α structure (PDB code: 1B53). The residues with ΔRR > 0.1 are *colored green*. V63, which was significantly affected in the methyl-directed TCS experiments (Yoshiura et al. 2010), is *colored dark green*. The molecular diagrams were generated with WebLab Viewer Pro (Molecular Simulations, Inc.)

As in previously reported methyl-directed TCS experiments (Yoshiura et al. 2010), we performed control experiments, in which rHDL without CCR5 was added to MIP-1α, to observe the non-specific binding effects, such as interactions with lipids or the MSPE3 polypeptide. The difference in reduction ratios (ΔRRs), which represents the specific interaction between CCR5-rHDL and the MIP-1α variant, was calculated for each resonance, by subtracting the intensity reduction ratio in the control experiment from that in the TCS experiment. As a result, A10, C11, Y15, T16, N23, F24, Y28, F29, T31, S32, T44, R46, Q49, V50, C51, V59, and Q60 exhibited high ΔRRs (>0.1) (Fig. 2a). These residues are on the N-loop (Y15 and T16), the β-sheets (A10, C11, N23, F24,Y28, F29, T31, S32, T44, R46, Q49, V50, and C51), and the C-terminal helix (V59 and Q60), and form a continuous surface on the β-sheets and N-loop region around Q49 and the α-helix region around V59 (Fig. 2b).

### TCS experiments with the MIP-1α variant and CCR1-rHDL

**Fig. 3:**
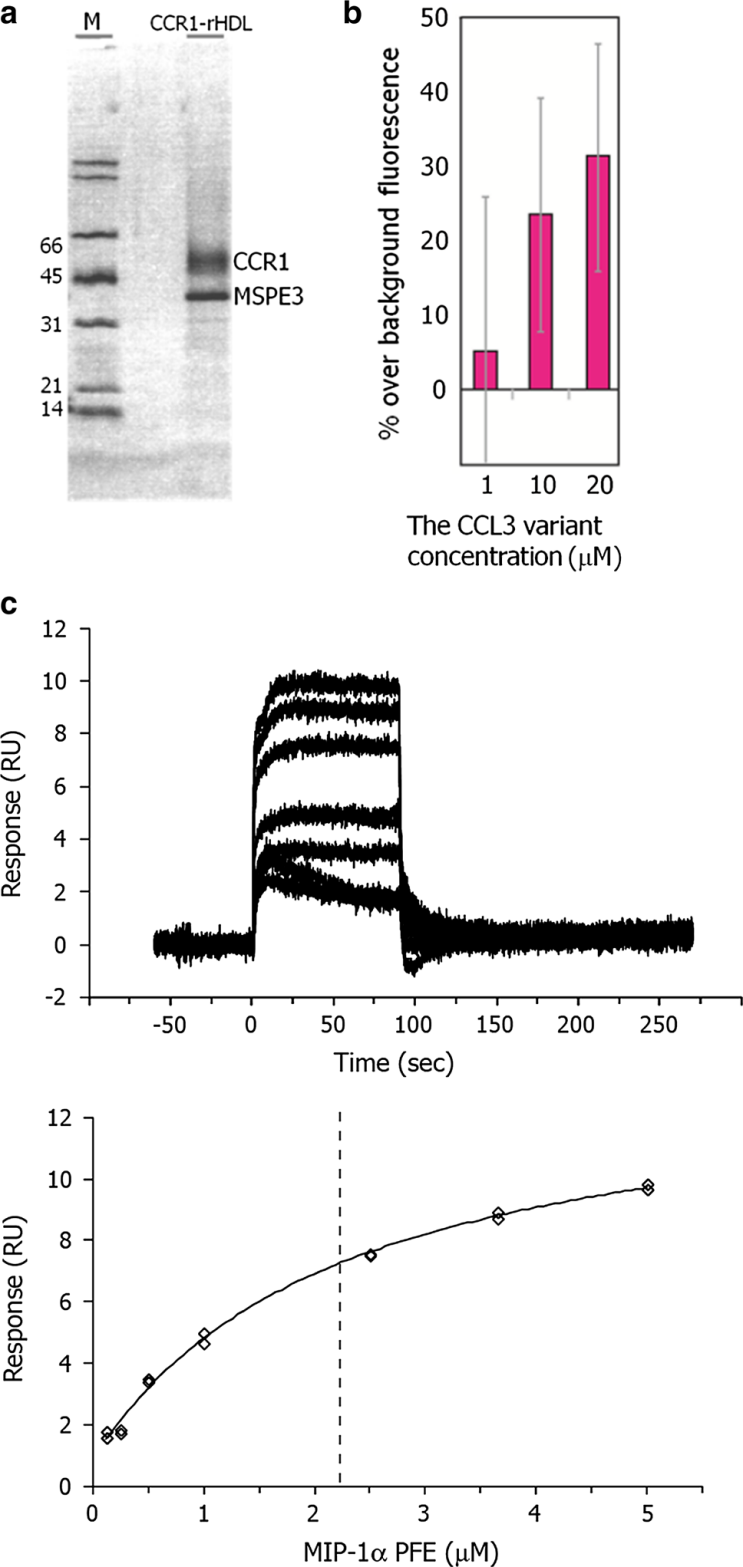
Characterization of CCR1-rHDL. **a** SDS-PAGE analysis of CCR1-rHDL. The purified CCR1-rHDL was loaded on a 15 % gel and silver-stained. **b** Eu-GTP binding to the complex of CCR1-rHDL and purified G protein, stimulated by the MIP-1α variant. Eu-GTP binding was measured after an incubation in the presence of various concentrations of the MIP-1α variant. Results are expressed as the percentage over the basal level of binding. Data represent the mean and SE of triplicate binding determinations from three separate, representative experiments. **c** SPR analyses of the interaction between the MIP-1α variant and CCR1-rHDL. The *upper panel* represents overlay plots of the sensorgrams obtained for the interaction between 0.125 and 5 μM of the MIP-1α variant and the immobilized CCR1-rHDL. The plots based on the steady-state method in SPR are shown in the *lower panel*. Each point is the average of 50 data points in the sensorgram, and the *error bars* are their standard deviations

We also performed the TCS experiments with the MIP-1α variant and CCR1-rHDL. CCR1 was prepared by the method previously developed for CCR5 (Yoshiura et al. 2010). CCR1, with a C-terminal 1D4 epitope-tag, was expressed in SF9-insect cells. The plasma membrane of the Sf9 cells was partially purified by sucrose gradient ultracentrifugation, to remove the soluble impurities. CCR1 was solubilized by dodecyl β-d-maltopyranoside (DDM) and then immediately reconstituted into rHDL, using MSPE3 with the N-terminal His tag. CCR1 in rHDL (CCR1-rHDL) was purified by removing the non-reconstituted elements, using Ni affinity chromatography, and then subsequently removing the rHDL without CCR1, using 1D4 antibody affinity chromatography. The purity of CCR1-rHDL was >80 %, as judged from the SDS-PAGE pattern (Fig. 3a). Western-blotting analyses with the 1D4 antibody revealed that about 40 μg (1 nmol) of CCR1 reconstituted in rHDL were obtained from a 1 L Sf9 culture. The yield was about four times higher than that of CCR5, and the minor band, which was observed in the CCR5-rHDL preparation, was still observed but markedly decreased (Yoshiura et al. 2010), probably because the higher yield of CCR1 resulted in fewer impurities. In the electrophoresis analysis (Fig. 3a), the band of CCR1 was higher than that of CCR5 (Yoshiura et al. 2010), which may be due to the more basic character of CCR5 (calculated isoelectric points of CCR1 and CCR5 are 8.8 and 9.45, respectively).

To examine whether CCR1-rHDL retains signal transduction activity, the GDP–GTP exchange assay was performed, using the P8A/F13Y/E67Q variant of MIP-1α, which exists as a monomer at pH 6.0 and stimulates the signaling by CCR5 (Yoshiura et al. 2010). The GDP–GTP exchange process on the Gα subunit was monitored by measuring the binding of non-hydrolyzable GTP modified with fluorescent europium. The intensity of the fluorescence was increased, dependent on the concentration of the MIP-1α variant, suggesting that CCR1-rHDL was activated by the MIP-1α variant and induced the GDP–GTP exchange on the Gα subunit (Fig. 3b).

We applied SPR to examine the binding between the MIP-1α variant and CCR1-rHDL. As a result, the responses were observed upon the addition of the MIP-1α variant to CCR1-rHDL immobilized on the sensor chip, and the estimated dissociation constant was ~2 μM (Fig. 3c). The sensorgrams were recorded ~12 h after the immoblilization of CCR1-rHDL, and the maximum response was comparable to that calculated from the amount of immobilized CCR1-rHDL, suggesting that most of the CCR1-rHDL retained the ligand binding activity for at least ~12 h.

**Fig. 4:**
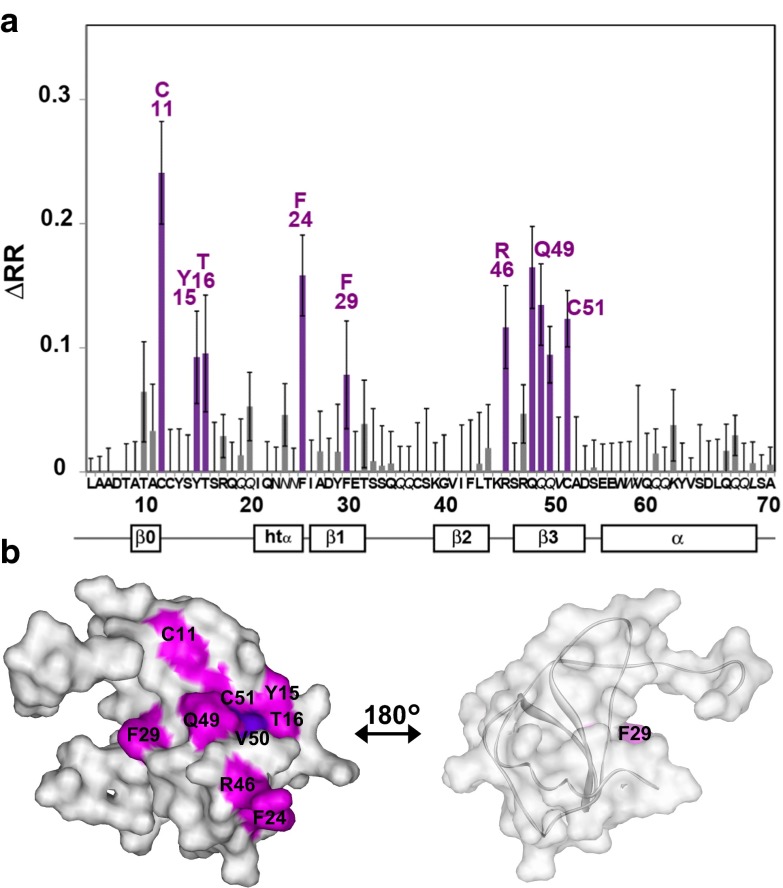
CCR1-binding site on MIP-1α determined with the TCS method along with Co-ANAFOR. **a** Plot of the difference in the reduction ratio (ΔRR) signal intensities originating from the amide groups in the amide-directed TCS experiments with MIP-1α and CCR1-rHDL. Cross-peaks with ΔRR > 0.07 and <0.07 are *colored green* and *gray*, respectively. The *error bars* represent the experimental errors, calculated from the root sum square of (noise level/signal intensity) in the four spectra, with and without irradiation in the main and control experiments. Sidechains are denoted in *italics*. **b** Mapping of the affected residues in the TCS experiments with CCR1-rHDL on the MIP-1α structure (PDB code: 1B53). The residues with ΔRR > 0.07 are *colored magenta*. V50, which was significantly affected in the methyl-directed TCS experiments (see Fig. S1 in Online Resource), is *colored dark purple*. These molecular diagrams were generated with WebLab Viewer Pro (Molecular Simulations, Inc.)

In the methyl-directed TCS experiments of CCR1-rHDL and MIP-1α, the resonances from V50 exhibited high ΔRRs (>0.1) (Fig. S1 in Online Resource 1). In the amide-directed TCS experiments along with Co-ANAFOR, C11, Y15, T16, F24, F29, R46, Q49, and C51 exhibited high ΔRRs (>0.07) (Fig. 4a). These residues are on the N-loop (Y15 and T16) and the β-sheets (C11, F24, F29, R46, Q49, and C51), and form a continuous surface (Fig. 4b).

## Discussion

Although the NMR measurement time for the CCR1 and CCR5 samples was limited to 24 h due to their low stability, even in the lipid bilayer environment of rHDL, the application of Co-ANAFOR to the amide-directed TCS experiments of MIP-1α with CCR1 and CCR5 revealed that the residues on the β-sheet and the N-loop region of MIP-1α around Q49 are in close proximity to CCR1 and CCR5 (Figs. 2, 4). As described previously (Yoshiura et al. 2010), the interactions observed in the TCS experiments were those of monomeric MIP-1α, CCR1, and CCR5 that retain their signal transduction activities, although glycosaminoglycans were absent and the sulfonation state of the tyrosine residues in CCR1 and CCR5 is unknown. The binding interfaces are generally consistent with those identified in the previously reported mutational studies on MIP-1α (Laurence et al. 2000, 2001; Bondue et al. 2002; Proudfoot et al. 2003), which shares 68 % sequence identity with MIP-1α, and the two-step binding mode proposed in our previous NMR studies (Kofuku et al. 2009) (See text in the Online Resource 1 for details).

**Fig. 5:**
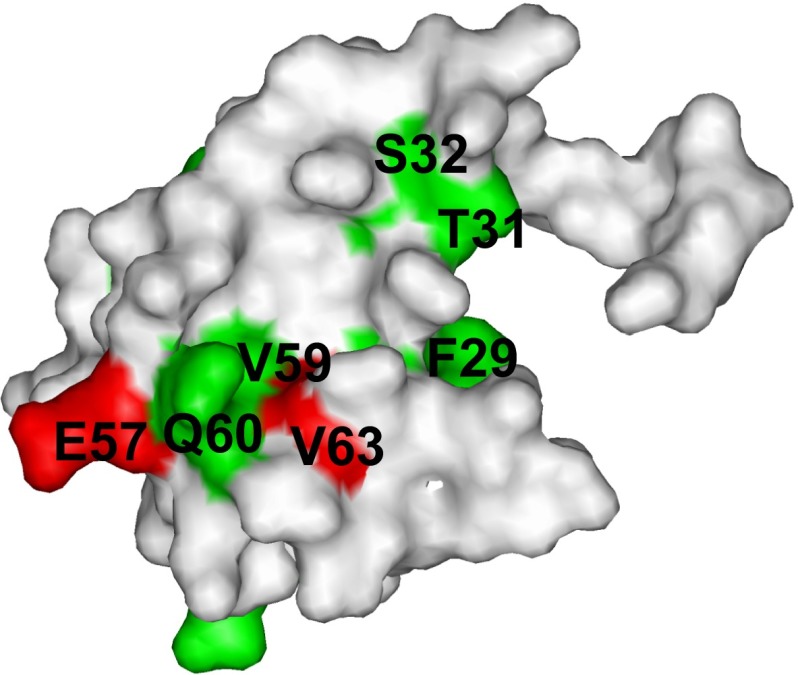
Mapping of the MIP-1α resides that are substituted with non-synonymous amino acids in reported single nucleotide polymorphisms (*red*). The residues in the CCR5-binding interface determined by the TCS experiments (Fig. 2) are *colored green*

The distinct expression patterns of CCR1 and CCR5, which were previously observed in the pattern II and pattern III MSs, suggested that CCR1 and CCR5 play different roles in inflammation (Mahad et al. 2004). Therefore, genetic analyses of CCR1 and CCR5 would provide clues toward elucidating how CCR1 and CCR5 affect the progress of MS and other autoimmune diseases. In addition, both CCR1 and CCR5 bind to several C–C type chemokines, such as MIP-1α, MIP-1β, and RANTES, and thus genetic analyses of each chemokine–chemokine receptor interaction are also important. A comparison of the CCR1- and CCR5-binding sites on MIP-1α revealed that the residues in the α-helix region around V59 are only involved in the CCR5-binding site (Fig. 5). This region includes E57 and V63, which are substituted in several reported single nucleotide polymorphisms (SNPs) with non-synonymous amino acids (Modi et al. 2006; Yoshiura et al. 2010). Therefore, these SNPs would specifically affect the CCR5–MIP-1α interaction, and the genetic influence of these SNPs in MS or other autoimmune diseases would provide clues toward elucidating how the CCR5–MIP-1α interaction affects the progress of autoimmune diseases (Mahad et al. 2004).

Although there are 28 C–C chemokines that share a similar fold, only nine and seven C–C chemokines, including MIP-1α, reportedly bind to CCR1 and CCR5, respectively (Allen et al. 2007). This ligand selectivity of chemokines plays important roles in the complex regulation of immune responses (Devalaraja and Richmond 1999; Mantovani 1999; Allen et al. 2007). Whereas most of the residues on the binding interfaces are highly conserved between the chemokines, three residues in the center of the binding site, A10, F29, and Q49, are not conserved except for MIP-1α and RANTES, which reportedly bind to both CCR1 and CCR5 (Fig. S2 in Online Resource 1). Therefore, the binding specificity would be at least partly due to the conservation of these residues.

## Electronic supplementary material

Supplementary material 1 (PDF 453 kb)
