# Supplementary material for: Elucidation of the CCR1- and CCR5-binding modes of MIP-1α by application of an NMR spectra reconstruction method to the transferred cross-saturation experiments
Source: J Biomol NMR. 2015 Oct 15;63(4):333–40. doi: 10.1007/s10858-015-9992-x (PMC4662715; doi:10.1007/s10858-015-9992-x)
Supplement: Supplementary file 1 — Supplementary material 1 (PDF 453 kb) [file 10858_2015_9992_MOESM1_ESM.pdf]

Online Resource 1:

“Elucidation of the CCR1- and CCR5- binding modes of MIP-1α by application of an NMR spectra reconstruction method to the transferred cross-saturation experiments”

Journal of Biomolecular NMR

Chie Yoshiura[1*], Takumi Ueda[1,2*], Yutaka Kofuku[1], Masahiko Matsumoto[1,3], Junya Okude[1], Keita Kondo[1], Yutaro Shiraishi[1], and Ichio Shimada[1]

[1]Graduate School of Pharmaceutical Sciences, The University of Tokyo, Hongo, Bunkyo-ku, Tokyo 113-0033, Japan

[2]Precursory Research for Embryonic Science and Technology, Japan Science and Technology Agency, Chiyoda-ku, Tokyo 102-0075, Japan

[3]Japan Biological Informatics Consortium, Aomi, Koto-ku, Tokyo, 135-8073 Japan

*These authors equally contributed to this work.

E-mail address of the corresponding author: shimada@iw-nmr.f.u-tokyo.ac.jp

Table of Contents

Text. Comparison of the proposed chemokine-chemokine receptor binding mode with previously proposed modes

Fig. S1. Methyl-directed TCS experiments for the determination of the CCR1-binding site on MIP-1α.

Fig. S2. Conservation of the residues in the CCR5-binding interface of MIP-1α.

Text

Comparison of the proposed chemokine-chemokine receptor binding mode with previously proposed modes

In previously reported mutational studies on MIP-1β, which shares 68 % sequence identity with MIP-1α, the N-loop and the 40's loop of MIP-1β were proposed to be important for the binding to CCR5 (Laurence et al., 2000; Laurence et al., 2001; Bondue et al., 2002; Proudfoot et al., 2003). V59 and V63, in the C-terminal helix, were identified as the residues in close proximity to CCR5 in the previously reported methyl-directed TCS experiments (Yoshiura et al., 2010), although the overall binding mode was unknown. The N-loop, the 40's loop, and the C-terminal helix are included in the binding interface determined by the present TCS experiments (Fig. 2). For V50 and V63, only one of the amide and methyl groups were affected by irradiation in the TCS experiments with CCR5-rHDL (Fig. 2), probably because the others are further away from the surface and thus the saturation is not efficiently transferred.

A two-step/two-site binding model has been proposed for the interaction between chemokines and chemokine receptors (Crump et al., 1997), and we have previously provided structural evidence that supports this model in the SDF-1α-CXCR4 interaction (Kofuku et al., 2009). In this model, two independent interactions are

hypothesized as follows: multiple regions throughout the chemokine interact with the extracellular region of the chemokine receptor, and subsequently the chemokine N terminus interacts with the chemokine receptor transmembrane region to trigger receptor activation. In the case of CCR5 and MIP-1α, the N-terminus of MIP-1α was proposed to only marginally contribute to the stabilization of the complex, because the N-terminal deletion mutant of MIP-1β can inhibit CCR5 with an inhibition constant similar to that of the wild type (Laurence et al., 2000). Therefore, they would exist in equilibrium between the major 1st step and the minor 2nd step, and the 1st step would be observed in the present TCS experiments. This is consistent with the TCS results, in which the ΔRR values of most of the N-terminus residues were < 0.1 (Fig. 2).

Crystal structures of other chemokine-chemokine receptor complexes, CXCR4-vMIPII and US28-CX3CL1, have recently been solved (Burg et al., 2015; Qin et al., 2015). In these structures, residues on the β-sheet and the N-loop region of the chemokines are close to the chemokine receptors, as well as the CCR1-MIP-1α and CCR5-MIP-1α complexes. In contrast, the α-helix region around the residues corresponding to V59 of MIP-1α, which are only involved in the CCR5-binding site, is not close to the chemokine receptors in these crystal structures. In the case of MIP-1α, the α-helix region may bind to the N-terminal region of CCR5 that corresponds to the

region not observed in the crystal structures of CXCR4-vMIPII and US28-CX3CL1 complexes.

References

Bondue, A., Jao, S.C., Blanpain, C., Parmentier, M., and LiWang, P.J. (2002). Characterization of the role of the N-loop of MIP-1 beta in CCR5 binding. Biochemistry **41,** 13548-13555.

Burg, J.S., Ingram, J.R., Venkatakrishnan, A.J., Jude, K.M., Dukkipati, A., Feinberg, E.N., Angelini, A., Waghray, D., Dror, R.O., Ploegh, H.L., and Garcia, K.C. (2015). Structural biology. Structural basis for chemokine recognition and activation of a viral G protein-coupled receptor. Science **347,** 1113-1117.

Crump, M.P., Gong, J.H., Loetscher, P., Rajarathnam, K., Amara, A., Arenzana-Seisdedos, F., Virelizier, J.L., Baggiolini, M., Sykes, B.D., and Clark-Lewis, I. (1997). Solution structure and basis for functional activity of stromal cell-derived factor-1; dissociation of CXCR4 activation from binding and inhibition of HIV-1. EMBO J. **16,** 6996-7007.

Kofuku, Y., Yoshiura, C., Ueda, T., Terasawa, H., Hirai, T., Tominaga, S., Hirose, M., Maeda, Y., Takahashi, H., Terashima, Y., Matsushima, K., and Shimada, I. (2009). Structural basis of the interaction between chemokine stromal cell-derived factor-1/CXCL12 and its G-protein-coupled receptor CXCR4. J. Biol. Chem. **284,** 35240-35250.

Laurence, J.S., Blanpain, C., Burgner, J.W., Parmentier, M., and LiWang, P.J. (2000). CC chemokine MIP-1 beta can function as a monomer and depends on Phe13 for receptor binding. Biochemistry **39,** 3401-3409.

Laurence, J.S., Blanpain, C., De Leener, A., Parmentier, M., and LiWang, P.J. (2001). Importance of basic residues and quaternary structure in the function of MIP-1 beta: CCR5 binding and cell surface sugar interactions. Biochemistry **40,** 4990-4999.

Proudfoot, A.E., Handel, T.M., Johnson, Z., Lau, E.K., LiWang, P., Clark-Lewis, I., Borlat, F., Wells, T.N., and Kosco-Vilbois, M.H. (2003). Glycosaminoglycan binding and oligomerization are essential for the in vivo activity of certain chemokines. Proc. Natl. Acad. Sci. **100,** 1885-1890.

Qin, L., Kufareva, I., Holden, L.G., Wang, C., Zheng, Y., Zhao, C., Fenalti, G., Wu, H., Han, G.W., Cherezov, V., Abagyan, R., Stevens, R.C., and Handel, T.M. (2015). Structural biology. Crystal structure of the chemokine receptor CXCR4 in complex with a viral chemokine. Science **347,** 1117-1122.

Yoshiura, C., Kofuku, Y., Ueda, T., Y., M., Yokogawa, M., Osawa, M., Terashima, Y., Matsushima, K., and Shimada, I. (2010). NMR analyses of the interaction between CCR5 and its ligand using functional reconstitution of CCR5 in lipid bailayers. J. Am. Chem. Soc. **132,** 6768-6777.

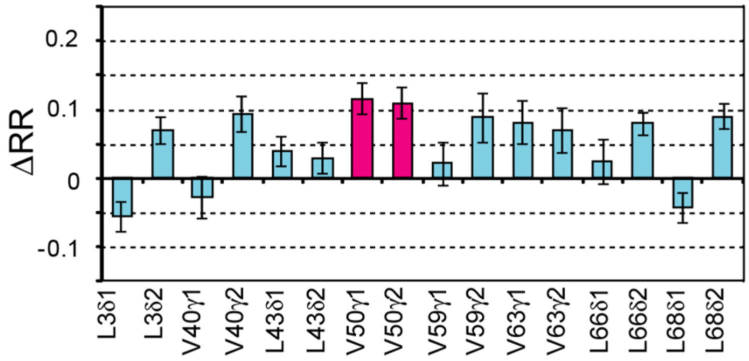

Fig. S1 Methyl-directed TCS experiments for the determination of the CCR1-binding site on MIP-1α. Plot of the difference in the reduction ratio (ΔRR) signal intensities originating from the leucine and valine methyl groups. Cross-peaks with ΔRR > 0.1 and < 0.1 are colored magenta and cyan, respectively. Error bars represent signal to noise ratios.

a

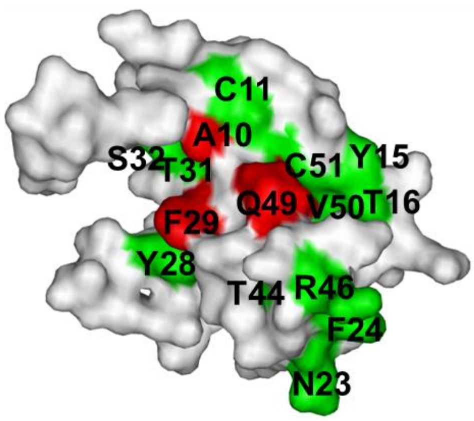

b

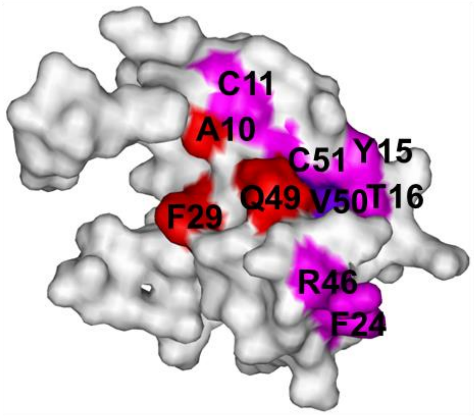

c

Chemokines	CCR1 binding	CCR5 binding	A10	F29	Q49
CCL3 (MIP-1α)	○	○	A	F	Q
CCL4 (MIP-1β)	○	○	A	Y	Q
CCL5 (RANTES)	○	○	P	F	Q
CCL1	-	-	R	R	E
CCL2	-	-	T	R	E
CCL7	○	-	T	R	E
CCL8	-	○	T	R	E
CCL11	-	○	T	R	D
CCL13	○	-	T	V	E
CCL14	○	○	E	Y	S
CCL15	○	-	D	F	Q
CCL16	○	○	T	-	E
CCL17	-	-	E	Y	A
CCL19	-	-	D	L	Q
CCL20	-	-	D	Q	S
CCL22	-	-	V	Y	E
CCL23	○	-	D	F	R
CCL24	-	-	P	L	Q
CCL25	-	-	D	Q	K
CCL26	-	-	T	F	K
CCL27	-	-	A	Q	-

Fig. S2. Conservation of the residues in the CCR5-binding interface of MIP-1α. (a), (b). Mapping of the A10, F29, and Q49 residues on the MIP-1α structure (red). (c). Comparison of the amino acid sequences that correspond to A10, F29, and Q49 of MIP-1α. The CCR5-binding site in (a) and the CCR1-binding site in (b), which were determined by the TCS experiments (Figures 2 and 4), are colored green and magenta, respectively.
